# Dynamic Response of Masonry Structures to Temperature Variations: Experimental Investigation of a Brick Masonry Wall

**DOI:** 10.3390/s24237573

**Published:** 2024-11-27

**Authors:** Daniele Pellegrini, Alberto Barontini, Nuno Mendes, Paulo B. Lourenço

**Affiliations:** 1 Institute of Information Science and Technologies “A. Faedo”, ISTI–CNR, 56124 Pisa, Italy; daniele.pellegrini@isti.cnr.it; 2 ISISE, ARISE, Department of Civil Engineering, University of Minho, 4800-058 Guimarães, Portugal; albe.barontini@gmail.com (A.B.); pbl@civil.uminho.pt (P.B.L.)

**Keywords:** masonry structures, dynamic properties, thermal loads, temperature variations, structural health monitoring

## Abstract

Structural health monitoring (SHM) is essential for preserving historical and modern infrastructure by tracking dynamic properties such as frequencies and mode shapes. Changes in these properties can indicate structural damage, but environmental factors like temperature can also cause similar variations, complicating damage detection. This study investigates from an experimental point of view the effect of temperature on the dynamic behaviour of masonry structures, focusing on a masonry wall subjected to thermal load variations within operational conditions. The experimental setup involved a masonry wall specimen tested at the Structural Laboratory of the University of Minho, Portugal. The mock-up was subjected to various boundary conditions and loading scenarios. The results showed that the natural frequencies of the masonry wall can be significantly influenced by temperature changes, variations strictly related to the boundary conditions and the stress acting on the mock-up. In contrast, mode shapes seem not to be affected by temperature variations. This study provides valuable insights into the temperature-induced variations in the dynamic properties of masonry structures, emphasising the need to consider environmental effects in SHM applications. By filtering out these environmental influences, more accurate damage detection and proactive maintenance strategies can be developed, enhancing the safety and longevity of both historical and modern structures.

## 1. Introduction

Structural health monitoring (SHM) plays a pivotal role in maintaining and preserving historical buildings and modern infrastructure. By continuously tracking the structure’s dynamic properties, such as frequencies and mode shapes, SHM represents a proactive measure to detect and mitigate potential issues, thereby averting structural failures or deterioration; indeed, significant changes in vibration measurements can serve as a damage indicator and therefore be used to alert those responsible for the structure’s safety [1].

One critical aspect of SHM is that changes in operational loadings and environmental conditions (e.g., temperature, humidity, phreatic level, sea waves, wind, occupancy, and live loads) can induce variations in the structure’s dynamic characteristics (especially in natural frequencies) that are comparable in magnitude to those caused by structural damage [2,3,4,5]. Therefore, it is essential to distinguish between normal variations and those resulting from deterioration, filtering out the influence of environmental parameters from the collected data.

Regardless of structural typology and material constituents, several monitoring campaigns denote temperature as the environmental parameter that can generate the most eigenfrequency variations [6,7,8]. Generally, in concrete, steel, and prestressed concrete structures, an increase in environmental temperature causes a decrease in natural frequencies [6,7,8]. Temperature instead affects the dynamic behaviour of monumental masonry buildings in a different way depending on the structural typology, boundary conditions, temperature interval range, and the presence or absence of stiffening elements [9]. For example, slender masonry constructions such as towers and bell towers typically experience a rise in frequency with increasing temperature [10,11,12,13,14]. This peculiar behaviour can be attributed to the temporary increase in structural stiffness caused by the closing of micro-cracks during thermal expansion. Differently, some historical masonry buildings retrofitted with tie rods exhibit negative frequency–temperature correlations for fundamental frequencies [15,16].

Table 1 summarises the main results obtained from the continuous monitoring campaigns mentioned above in terms of the maximum air temperature range recorded and the corresponding maximum variation in the first two frequencies tracked during the monitoring period. The various cases reported in the literature have been deeply examined in [9], to which the reader is referred to for further details.

Several studies have addressed the effect of high temperature on the physical and mechanical properties of masonry constituents, such as mortar, bricks, or stone blocks [17,18,19,20,21,22,23], although mainly for fire safety purposes. Nonetheless, a comprehensive understanding of the complex interaction of the ongoing phenomena at the composite structural element and building level, with specific attention paid to the influence on the dynamic behaviour in normal operational conditions, is still missing. To the authors’ knowledge, only two laboratory tests have investigated the correlation between the dynamic properties of masonry structures and temperature variations, focusing on a single typology (i.e., arches) and, in one case, on extreme temperatures. The effects of environmental variations on the dynamic characteristics of a brick masonry arch with gypsum mortar were studied in [24], conducting ambient vibration tests at three temperatures between 14 °C and 38 °C. The results indicated that the arch’s dynamics were highly sensitive to the test conditions, with heating causing somewhat unpredictable effects, comprising an increase in first and fourth frequencies by about 3% and a decrease in second and third frequencies by approximately 3.5%. Increased moisture content led to a general decrease in natural frequencies, but no significant changes were observed in the mode shapes. Altunişik et al. [25] investigated the modal properties of masonry arches subjected to 20 °C and 600 °C, analysing five specimens with different cross-sections through ambient vibration tests before and after heating. The natural frequencies of all specimens decreased by about 50–55% due to material degradation (especially compressive strength). This study also included a finite element (FE) numerical analysis using a macro-modelling approach. The components were modelled as linear elastic materials, and their Young’s moduli were calibrated through a model updating procedure to match the experimental frequencies.

The present paper aims to fill this gap in the current literature, investigating from the experimental point of view the three-dimensional dynamic behaviour of a small masonry wall subjected to shifts in thermal load within expected operational conditions and different realistic loading and boundary conditions.

In particular, the tests carried out in the present research allow for the identification of both trends, with the overall behaviour being governed by two main phenomena with opposite consequences, namely the deterioration at the material level and the confinement effect due to the interaction among structural elements. Furthermore, the research goal is to understand if it is possible to replicate the findings from continuous monitoring campaigns on masonry structures in the laboratory. If successful, this initial step will pave the way to plan subsequent systematic tests on different structural types and build a database for testing and applying, in a second step, a FE numerical procedure, investigated by the authors in [9,26], to calculate the frequencies of masonry structures in the presence of thermal loads.

The remainder of this paper is organised as follows. Section 2 describes the setup, the masonry mock-up employed in the experimental campaign, and the test sequence. Section 3 shows and discusses the results of each experimental test in terms of natural frequencies and mode shape variations. Finally, Section 4 summarises the main conclusions of this paper.

## 2. Experimental Setup

**Figure 1:**
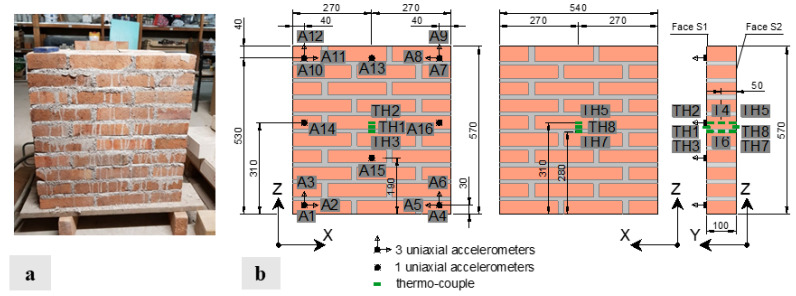
Masonry wall mock-up after construction (**a**); specimen dimensions (in mm) and the sensor layout (**b**).

**Figure 2:**
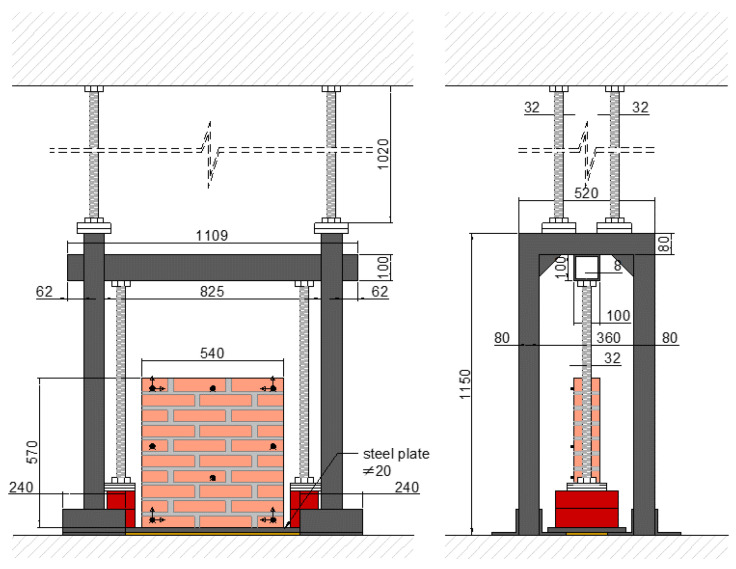
Experimental setup scheme (dimensions in mm).

**Figure 3:**
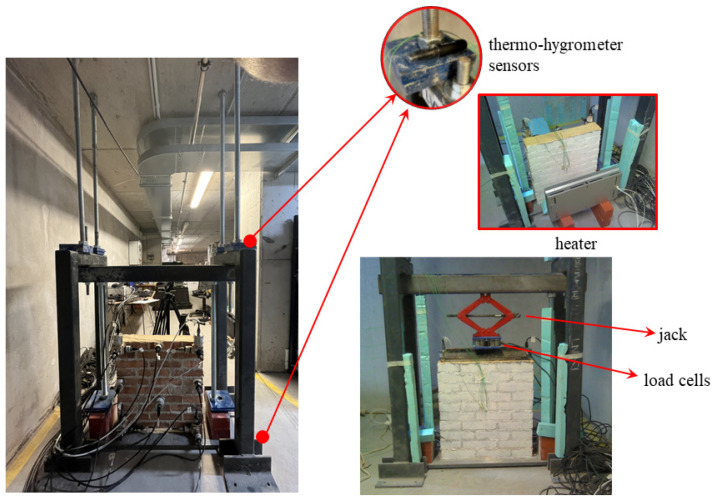
Experimental test T1 (**left**) and T3 (**right**).

The small masonry wall specimen under investigation was constructed and tested within the Structural Laboratory facilities at the University of Minho, located in Guimarães, Portugal. The specimen was assembled using premixed cement-based mortar joints, with a nominal compressive strength of 5 MPa, typically 10 to 20 mm thick, and ten rows of extruded solid clay brick units, with average dimensions of 200 × 100 × 45 mm^3^. The same bricks were already used in other experimental programmes at the same laboratory, for which extensive material characterisation tests exist [27,28]. The brick average compressive strength and Young’s modulus were 22.3 MPa and 9650 MPa. The wall featured a length of 540 mm, a height of 560 mm, and a thickness of 100 mm, as depicted in Figure 1. The mock-up was built outside the steel frame setup adopted for the tests (Figure 2 and Figure 3) and placed in it upon curing for more than 120 days. The specimens were fixed at the ground utilising a bi-component epoxy resin joint with a high adhesive capacity and a high resistance to temperature; a further layer of epoxy resin with high thermal resistance was used to rectify the upper face of the wall. High-strength ratchet straps secured the wall during transport to prevent damage.

The steel frame setup, seen Figure 2, comprises two identical portals connected through a square hollow section profile and a 20 mm thick steel plate used as the base for the mock-up. Threaded rods with 32 mm diameter were used to brace the setup, holding it against the concrete reaction slab to prevent rocking, sliding, and rotation.

Four distinct experiments were devised to encompass three main boundary conditions and types of actions, as outlined in Table 2 and described hereafter:

- T1 and T2 refer to tests in which the specimen was clamped at the base and subjected to increasing (mechanical heating) and decreasing (natural cooling) thermal loads on one side and, in the last stage, on both sides. These tests focused on the effects of the thermal variation on the masonry constituents and the composite wall, minimising the influence of other factors that may influence their behaviour. The heating on a single side recreates the typical condition of a façade wall with its thermal gradient, while the heating on both sides simulates the condition of an internal wall.
- T3 designates a test where the panel was clamped at the base with restricted top displacement along the Z direction. A scissor lift jack was used to prevent the mock-up top displacement, introducing an initial compression of about 5 kN. A stiff steel beam was employed to distribute the action and two load cells, located between the jack and the beam, as shown in Figure 3, which measured the corresponding reaction force. The specimen was subjected to increasing and decreasing thermal loads applied to the external faces so that the two sides had the same temperature at the recording time of the specimens’ vibrations. In this test, the temperature variations, investigated in T1 and T2, are combined with the effect of a vertical constraint. This simulates the interaction among elements in a real structure, causing an increase in the stresses under different thermal expansions.
- T4 represents a test with the mock-up clamped at the base and an increasing load applied to the top face by a scissor lift jack. A steel beam distributed the action on the specimen. Two load cells between the jack and the beam measured the applied load. Unlike the other tests, the specimen’s temperature, laboratory humidity, and temperature were kept constant throughout the experiment. This test aims to analyse the effect of a load cycle within the range experienced in the previous tests but under constant environmental conditions (average temperature 24 °C), investigating the influence on the natural frequencies in terms of shift at various load extents and residuals at the end of the cycle.

To identify the specimen’s dynamic properties over each test, sixteen high-sensitivity accelerometers were utilised: twelve PCB 393B12 with a frequency range of 0.15 to 1000 Hz, a sensitivity of 10,000 mV/g, a resolution of 8 μg, with an operating temperature range from −45 to +82 °C and weight of 0.21 kg, and four PCB 393B31 with a frequency range of 0.1 to 200 Hz, a sensitivity of 10 V/g, a resolution of 1 μg, with an operating temperature range from −26 to +65 °C and weight of 0.63 kg. The accelerometers were positioned on one side of the wall (face S1, see Figure 1). The decision to use such a dense sensor array was driven by the need to capture high-resolution modal properties, particularly mode shapes. To ensure a data acquisition window of at least 1000–2000 times the fundamental period of the wall [29], signals were sampled at 1000 Hz for a minimum duration of 300 s, resulting in 300,000 data points per channel. The temperature of the masonry panel was monitored using six thermocouples arranged as depicted in Figure 1: four thermocouples (two on each side) labelled TH2-TH3 and TH5-TH7 were placed inside the panel approximately 10 mm from the outer edge, one in a mortar joint, and one in a brick. Additionally, two thermocouples labelled TH4 and TH6 were positioned in the centre of the panel (50 mm deep in the brick and mortar joint, respectively). The last two, TH1 and TH8, were placed on external faces S1 and S2, slightly detached from the surface to record air temperature near it. Temperature was continuously recorded throughout each test at a sampling rate of 0.1 Hz. As described before, during tests T3 and T4, the wall top displacement (and the upward load) was prevented (applied) using a 3-ton scissor lift jack. The corresponding reaction force was measured, at a sampling rate of 1 Hz, by two load cells (RLC, maximum load capacity of 10 kN) located between the jack and the beam (Figure 3). The setup included two thermo-hygrometer sensors (model LASCAR EL-USB-2, measurement range of –35 °C to 80 °C and 0 to 100%RH, accuracy 0.45 °C and 3%RH) positioned at the base and the top of the frame setup, respectively (Figure 3), to monitor the surrounding environmental parameters (temperature and relative humidity), along with a thermo-camera (FLIR-T62101) to visualise the external thermal field within the specimen over each experiment. Temperature variations were induced by two infrared heaters acting individually on one side or simultaneously on both faces of the mock-up, each located 0.60 m away from the panel. Applying the thermal load on one or both faces of the specimen aims to simulate a thermal gradient throughout the wall thickness, which can influence its dynamic properties differently, unlike a uniform thermal load, as found numerically [9] and experimentally [15,16].

The temperature range applied in each test (not directly controllable as the heaters have no thermostat) is chosen to reproduce, in the mock-up, a uniform thermal field or a gradient temperature comparable to those listed in Table 1. Moreover, this range was limited to ensure that the temperature recorded in the proximity of the accelerometers was within their operating bounds to ensure their correct functioning and prevent the degradation or damage of the devices. The maximum temperature achieved during the experimental programme was recorded in T2 due to the heating of a single side of the wall (face S2) opposite to the location of the accelerometers.

## 3. Experimental Results

This section describes the results of each experimental test. In order to identify the dynamic properties of the masonry wall, all the signals sampled at 1000 Hz were recorded and collected in packages with a time duration greater than or equal to 300 s. Each signal was pre-processed by applying a fourth-order Butterworth bandpass filter [5–250] Hz, and then each dataset was analysed by employing two well-known and complementary methods implemented in the MACEC 3.3 software [30]: Frequency Domain Decomposition (FDD) and Stochastic Subspace Data Driven (SSI-dat) method. The SSI analysis was performed for an order model varying from 2 to 100 with a step increment of 2. The relative tolerance used in the stabilisation diagram to isolate stable poles from the noise modes is 0.01 for frequencies, 0.05 for damping, 0.01 for modal assurance criterion (MAC) [31], and 0.8 for modal phase collinearity (MPC) [31]. For further details on the dynamic identification process and the parameters listed above, the interested reader may refer to [30].

The results obtained by the two numerical procedures are identical in terms of frequencies and mode shapes; in the rest of the article, however, only the results relating to the SSI method will be shown for the sake of brevity and because this method also provides information relating to the damping ratio.

Regarding the effect of temperature on damping, it was not possible to draw consistent conclusions. No clear correlation between estimated damping ratios and temperature emerged during the experimental campaign. This is likely due to the higher uncertainties associated with identifying damping in ambient vibration tests compared to natural frequencies and mode shapes, reflected in estimation errors that may mask the effects of the temperature variation. Furthermore, the damping ratio does not represent a significant index for anomaly detection and damage localisation.

This issue is well documented in the literature and has been observed in various continuous dynamic monitoring campaigns [12,32,33,34]. Therefore, the relative results are not reported or discussed further; only the damping values estimated at the beginning of each test corresponding to the reference temperature are shown in Table 3, Table 4 and Table 5.

Section 3.1 summarises and compares the results of T1 and T2; Section 3.2 collects the outcomes of T3; and Section 3.3 describes the T4 results.

### 3.1. Test T1 and T2

As reported in Table 2, during tests T1 and T2, the wall specimen was clamped at the base and, otherwise, free to deform in any direction; the thermal load was applied only in correspondence with the face S2 (Figure 1). Before starting each test, a preliminary dynamic identification was performed at the reference temperature q_0_, defined as the environmental temperature measured at the beginning of each experiment. The results in terms of natural frequencies, damping, mode shapes, and MPC are summarised in Table 3.

**Table 3:** Specimen modal properties at the reference temperature (T1 and T2 test).

	Frequency f_i_ [Hz]	Damping [%]	MPC	Direction
Test T1, q_0_ = 23.27 °C				
Mode 1	43.06	3.01	1.00	Bending around X axis
Mode 2	140.76	3.78	0.89	Torsion around Z axis coupled to a shear deformation along X
Mode 3	148.27	2.28	0.90	Torsion around Z
Test T2, q_0_ = 23.30 °C				
Mode 1	42.32	3.02	1.00	Bending around X axis
Mode 2	138.86	3.17	0.92	Torsion around Z axis coupled to a shear deformation along X
Mode 3	146.55	3.17	0.80	Torsion around Z

**Figure 4:**
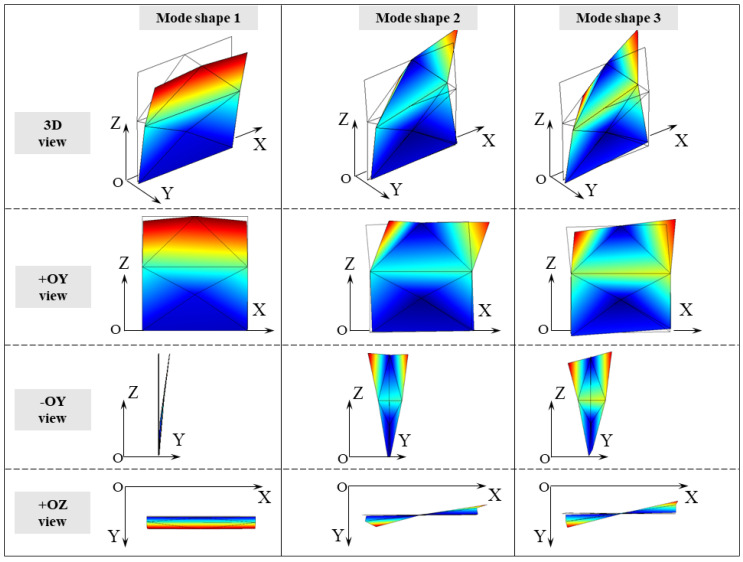
Test T1 and T2: experimental mode shapes at reference temperature.

Figure 4 sketches the first three mode shapes of the panel at the reference temperature for both experiments. They correspond to a bending mode along the Y direction (mode shape 1), a torsional mode shape around the *Z* axis (i.e., vertical axis) coupled with a bending mode in the XZ plane (mode shape 2), and a torsional mode shape around the vertical axis (mode shape 3). In the representation of the mode shapes, exclusively the measured degrees of freedom are presented. Therefore, in the second mode shape, the nodes located at half the height of the panel (XZ plane) appear stationary, but this is only because no accelerometers were placed on these nodes along the X direction.

**Figure 5:**
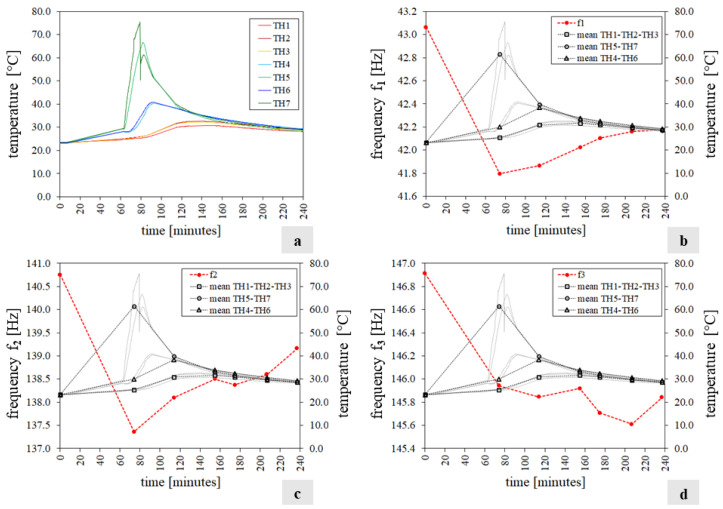
Test T1 results: (**a**) the temperature histories recorded by the thermocouples; (**b**–**d**) the specimen’s first three natural frequencies f_i_(q) (red line), the mean temperature values measured in the wall (black lines), and the temperature histories recorded by the thermocouples (grey lines).

**Figure 6:**
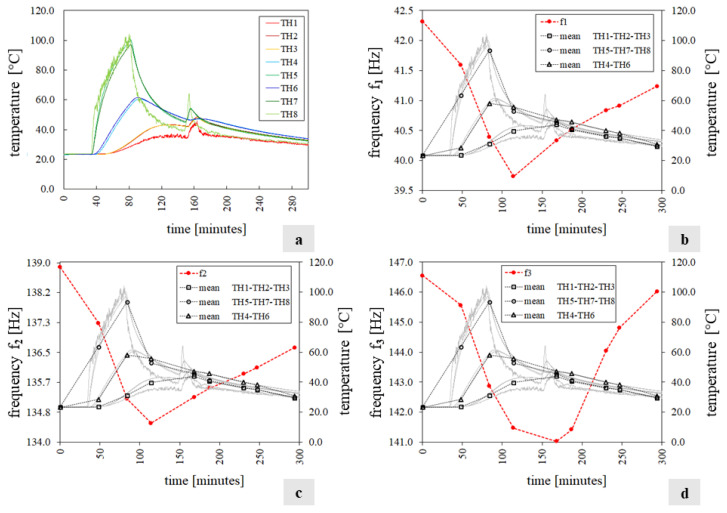
Test T2 results: (**a**) temperatures q measured by the thermocouples; (**b**–**d**) the mock-up’s first three natural frequencies f_i_(q) (red line), the average temperatures recorded (black line), and the temperature histories recorded by the thermocouples (grey lines).

Figure 5 and Figure 6 sum up the experimental results of tests T1 and T2, respectively, in terms of frequencies and temperature variations. Specifically in each figure, the chart labelled with “a” shows the temperatures q tracked by the thermocouples during the test while the first three frequencies f_i_(q) (i = 1…3) of the mock-up, estimated through the experiment, are depicted in diagrams b, c, and d and reported in Table A1 and Table A2 (Appendix A).

Each chart (b, c, and d) displays the frequency trend with a dashed red line. At the same time, the temperature q of the exposed face S2, unexposed face S1, and specimen’s core is represented by black lines with circular, box, and triangle markers, respectively. These quantities are calculated as the average temperatures recorded by the corresponding thermocouples. Note that during test T1, the TH8 thermocouple did not work (Figure 5a), and in test T2, after 160 min, a second infrared heater was used to increase the unexposed face’s temperature (S1) and make the specimen’s temperature field approximately uniform (Figure 6a).

The last row of Table A2 reports the values of the mock-up’s frequencies measured the day after before changing the boundary conditions and performing the T3 test described in Section 3.2.

**Figure 7:**
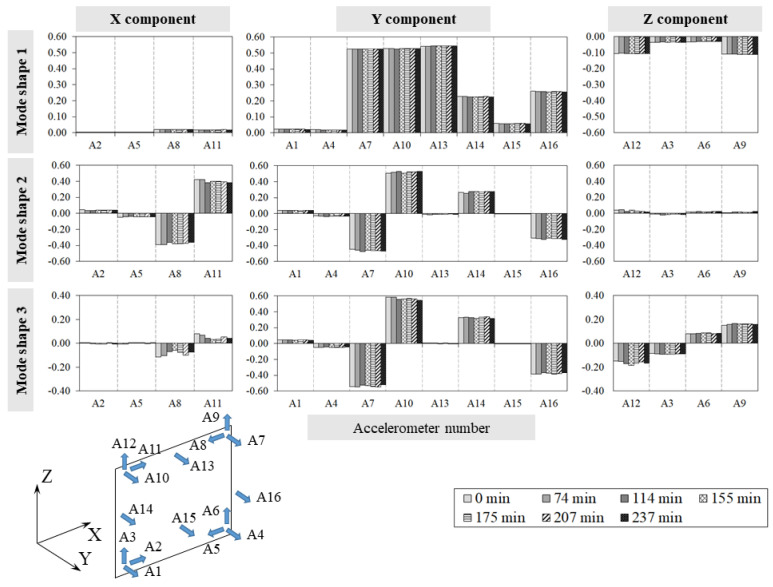
Test T1: mode shapes ϕ_i_ single components’ variations.

**Figure 8:**
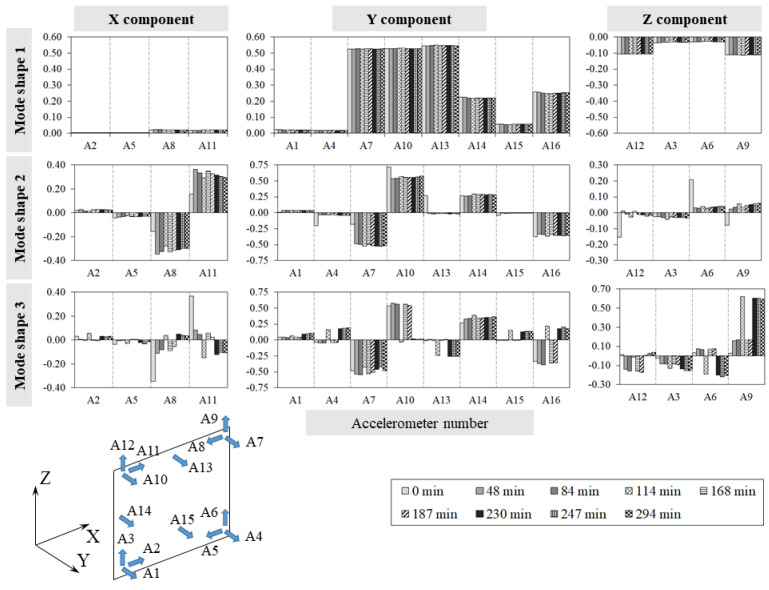
Test T2: mode shapes ϕ_i_ single components’ variations.

Figure 7 and Figure 8 collect, for every experiment, the single component variations along the three directions, X, Y, and Z, for each mode shape at different temperatures, while Table A3 and Table A4 (Appendix A) summarise the MAC values calculated during the tests between the specimen’s first three mode shapes ϕ_i_ at temperature q and the corresponding ones ϕ_i0_ at the reference temperature q_0_.

**Figure 9:**
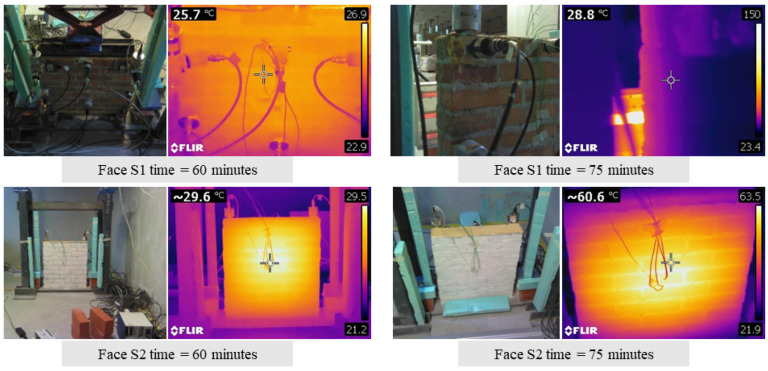
Test T1: wall temperature field detected through the thermo-camera.

Finally, Figure 9 shows an example of the temperature field sensed by the thermo-camera at two different experiment instants during test T1.

The analysis of the experimental results allows us to make the following considerations:

- Throughout both T1 and T2, the temperature in the bricks and adjacent mortar joints is approximately the same as highlighted in Figure 5a and Figure 6a by the curve pairs related to TH2-TH3, TH4-TH6, and TH5-TH7 in Figure 9.
- The maximum temperature difference between the exposed and unexposed face is about 40 °C and 60 °C in tests T1 and T2 (obtained before using the second infrared heater), respectively.
- The temperature field of the exposed face S2 seems to decrease uniformly from the centre of the wall to the edges (Figure 9).
- In the case of T1, the first two frequencies, f_1_ and f_2,_ decrease as the temperature increases while they rise and tend towards their initial values at the reference temperature as the temperature reduces.
- The third frequency, f_3_, estimated during the T1 test, appears to decrease regardless of the heat load trend, although when the temperature decreases, it reduces less abruptly.
- During the T2 experiment, the three frequencies tracked show a decreasing trend as the temperature increases and increasing trend as the thermal load decreases.
- In the case of T1, the drop in the first frequency has a maximum of about 3.0%, while the second and third frequencies have a reduction of 2.4% and 1.8%, respectively (Table A1).
- In regard to the T2 test, the maximum first frequency reduction is about 6%, while it is about 3–4% for the other two frequencies (Table A2).
- The single displacement components, except the third mode monitored during T2, and the mode shapes are not significantly influenced by the temperature variations, as shown in Figure 7 and Figure 8 and highlighted by Table A4 and Table A5, where the minimum MAC value is 0.94. This fact confirms that the vibration modes are less sensitive to temperature variations than the frequencies, as already highlighted by some experimental works [10,15,16].
- The MPC coefficient related to each mode shape is greater than 0.8, and its value does not suffer significant variations, which suggests the absence of damage in the specimen [35], as confirmed by a visual survey.

**Figure 10:**
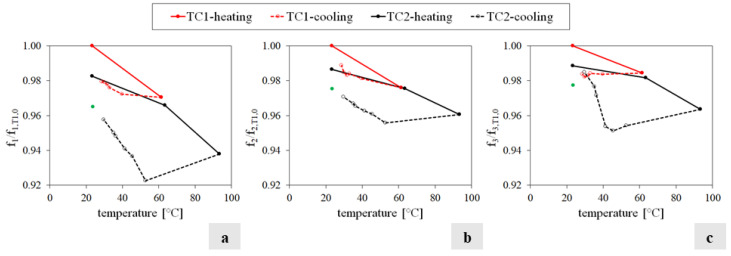
Comparison of the frequency f_1_ (**a**), f_2_ (**b**) and f_3_ (**c**) trend of T1 (red line) and T2 (black line) experiments versus temperature. The continuous lines represent the results obtained during the heating phase and the dashed lines provide the results of the cooling phase. The green dots represent the frequencies obtained the day after before performing the T3 test.

Finally, Figure 10 compares the wall’s frequency variations estimated over the T1 and T2 experiments for each temperature value. In each diagram, the horizontal axis traces the maximum temperature of the wall’s exposed face, while the vertical axis reports the ratio between the frequency f_i_ (i = 1…3) estimated at the temperature q and the value of the corresponding frequency f_i,T1,0_ (i = 1…3) of the T1 test, evaluated at the reference temperature q_0_. The red line represents the T1 results, while the black line stands for the T2 outcomes; the continuous lines refer to the frequency values estimated during the heating phase of the specimen, while the dashed lines represent the cooling phase. The green dots represent the mock-up’s frequencies estimated the day after, before performing the T3 test, thus with the same boundaries and comparable environmental conditions (Table A2).

The analysis of Figure 10 allows us to draw the following remarks:

- The relationship between frequencies and temperatures is sensitive to the direction of the thermal variation, presenting two trends: one during heating and one during cooling.
- In each cycle, two significant frequency drops are observed: a larger drop during the heating phase, particularly near the maximum temperature, and a residual drop following the cooling phase, at the end of each thermal cycle, although the specimen temperature is close to the reference one.
- During the second cycle, a time lag emerges between the maximum temperature and the minimum values of the natural frequencies.
- During the second cycle, the relationship between the variation in frequencies and temperature appears to be bi-linear, with the first stage being consistent with the decreasing trend during the cooling phase of T1 and the second stage consistent with the original heating phase of T1 for higher values of temperature that were not experienced in the first test.

Excluding a variation in the specimen’s mass or a change in the boundary conditions during T1 and T2, it is reasonable to attribute this frequency decrement to a stiffness reduction. No visible damage was observed in the mock-up during or after the tests, suggesting that this reduction in stiffness likely occurred at the material level. The detrimental effect of temperature on stiffness has been experimentally demonstrated in several studies concerned with the fire resistance of masonry and cementitious materials, although mostly focusing on higher temperature ranges. The increase in temperature induces physical and chemical alterations in their microstructure, including mineralogical transformations and the formation of micro-cracks due to thermal-induced dilatations, especially in the areas of contacts between components that undergo different deformations, as in the mortar joints [20,22,23]. As the thermal load diminishes, the fractures close, increasing mock-up stiffness and, consequently, its natural frequencies. However, the specimen never recovers its original stiffness, presenting a residual downshift in the natural frequencies.

### 3.2. Test T3 Results

Test T3 was conducted on the wall clamped at the base with restricted top displacement along the Z direction.

Before the experiment started, a preliminary dynamic identification at the reference temperature q_0_ was carried out. The outcomes in terms of natural frequencies, damping ratio, mode shapes, and MPC are presented in Table 4.

**Table 4:** Test T3: specimen’s dynamic characteristic at the reference temperature.

	Frequency f_i_ [Hz]	Damping [%]	MPC	Direction
Test T1, q_0_ = 23.74 °C				
Mode 1	36.66	3.48	1.00	Bending around X axis
Mode 2	117.27	2.35	0.88	Torsion around Z axis coupled to a shear deformation along X
Mode 3	122.73	1.21	0.96	Torsion around Z

The values of the three frequencies estimated at the reference temperature (i.e., initial point of the cycle) are lower than those recovered in the T1 and T2 tests (Table 3) likely due to the added masses during the T3 experiment consisting of the steel beam, the load cells, and the jack placed on the upper face of the specimen. This effect was more relevant than the effect of the initial compression introduced by the flat jack and expected to induce a slight increase in the frequencies.

**Figure 11:**
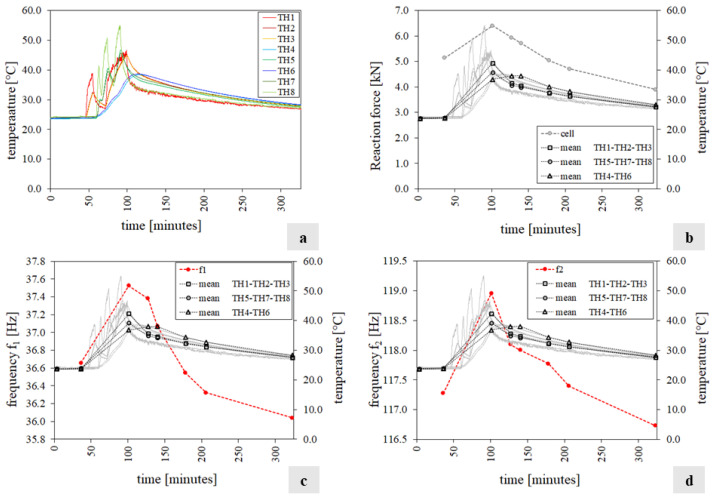
Test T3 results: (**a**) the mock-up temperature field; (**b**) reaction force (grey dot line) and the average temperatures collected (black line); (**c**,**d**) the mock-up’s first two natural frequencies f_i_(q) (i = 1…2) (red line), the average temperatures (black line), and the temperature histories recorded by the thermocouples (grey lines).

Figure 11 summarises the test outcomes in terms of (a) temperature histories q, (b) temperatures and reaction force measured by the load cells, and (c,d) temperatures and the specimen’s first two frequency variations. Note that the third frequency had no shifts, so the results are not reported. In particular, Figure 11c,d show the frequency trend with a dashed red line, while the temperature q of the exposed face S2, unexposed face S1, and specimen’s core is represented by black lines with circular, box, and triangle markers, respectively. These quantities are calculated as the average temperatures recorded by the corresponding thermocouples.

In this case, differently from tests T1 and T2, the first two frequencies increase as the temperature increases with a maximum of 2.37% and 1.44%, respectively (Table A5, Appendix A), while they drop as the temperature decreases, showing a maximum reduction of 1.68% and 0.46% (Table A5). This phenomenon can be explained by the fact that as the temperature rises, the mock-up expands and undergoes a precompression induced by the top restrain that prevents deformation along the vertical direction. This is reflected in an increment in the recorded load with temperature. The micro-cracks in the mortar joints close, leading to a temporary increase in the specimen’s stiffness and, consequently, in its frequency.

**Figure 12:**
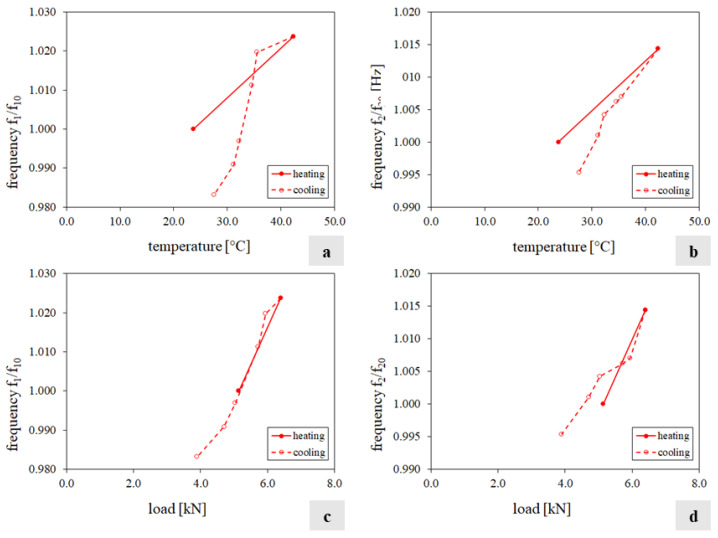
Test T3 results: (**a**,**b**) variation in frequencies f_1_ and f_2_ versus the temperature value of S2 face; (**c**,**d**) variation in frequencies f_1_ and f_2_ versus the reaction force.

Figure 12a,b show the wall’s frequency variations versus the temperature value. In each diagram, the horizontal axis traces the maximum temperature of the S2 face, while the vertical axis reports the ratio between the frequency f_i_ (i = 1…2) estimated at the temperature q and the corresponding value f_i0_ estimated at the reference temperature q_0_. Figure 12c,d show the frequency variations versus the reaction force. In all four charts, the solid line refers to the heating phase, while the dashed line refers to the cooling phase. The relationship between the variation in frequencies and temperature, identified in tests T1 and T2, with a different trend during heating and cooling, is here confirmed, although with an opposite tendency. A linear relationship between variation in frequencies and load emerges.

It is noted that at the end of the test, although the temperature is higher than the reference q_0_, the frequency is lower than its starting value along with the reaction force value. This phenomenon can be due to the evolution of physical and chemical microstructural alterations under the interaction of the thermal variation and the load imposed by the restricted top displacement. This is reflected in a slight reduction in the stiffness that increases the mock-up’s deformability, leading to a reduction in the measured load.

**Figure 13:**
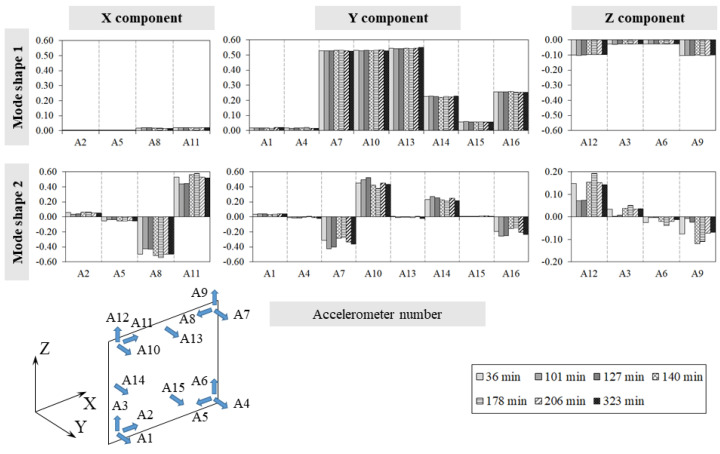
Test T3: single component variations in the two mode shapes.

Finally, Figure 13 depicts the evolution of the individual displacement components of the mode shapes during the experiment. Although Table A5 supports the idea that mode shapes are generally less affected by temperature changes than frequencies, the individual displacement components of the second mode appear to be influenced by local thermal fluctuations, potentially linked to the development of micro-cracks in some areas.

### 3.3. Test T4 Results

The last test, T4, was conducted on the wall clamped at the base, and an increasing load was applied to the top face by a scissor lift jack under constant environmental conditions.

Before the experiment started, a preliminary dynamic identification was carried out, and the results in terms of natural frequencies, damping ratio, mode shapes, and MPC are outlined in Table 5. During this acquisition, steel beam, load cells, and jack were left on the upper face of the specimen without inducing any compression on it.

**Table 5:** Test T4: specimen’s reference dynamic characteristic.

	Frequency f_i_ [Hz]	Damping [%]	MPC	Direction
Test T1, q_0_ = 23.74 °C				
Mode 1	25.83	5.83	1.00	Bending around X axis
Mode 2	114.72	1.25	1.00	Torsion around Z axis coupled to a shear deformation along X

**Figure 14:**
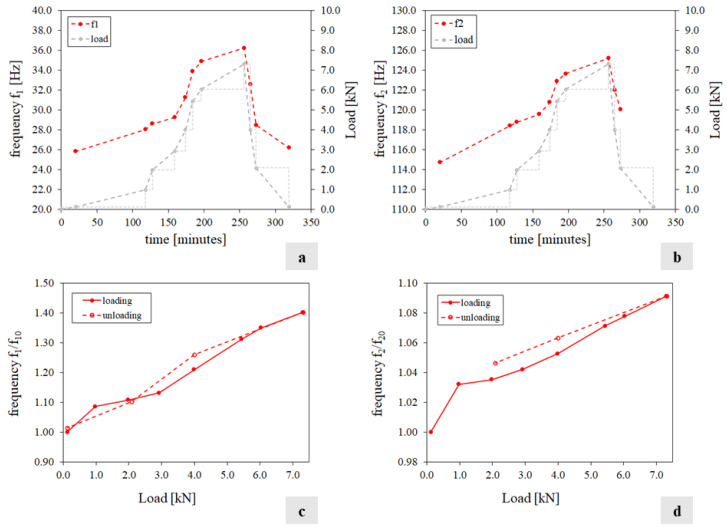
Test T4 results: (**a**,**b**) frequencies f_1_, f_2_, and the applied load variation; (**c**,**d**) frequencies f_1_ and f_2_ versus the applied load.

**Figure 15:**
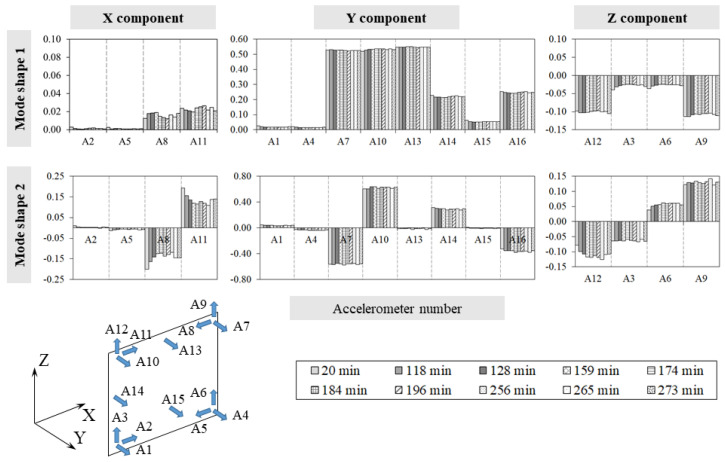
Test T4: mode shapes single components’ variations.

Figure 14 summarises the test results in terms of frequencies and load time histories (charts a and b) and the ratio between the frequency f_i_ (i = 1…2) estimated at the temperature q and the corresponding value f_i0_ estimated at the reference temperature q_0_ versus the applied load (charts c and d). Figure 15 shows the variation in the individual displacement components of the mode shapes over the experiment.

From the analysis of the results, it is clear that during the loading phase, the two frequencies tracked increase with a maximum variation equal to 40% and 9%, respectively (Table A6, Appendix A). Regarding the mode shapes, the applied loads do not affect the eigenvectors globally (Table A6), even if the single components of the second mode shape along the X and Z directions present small fluctuations under different levels of load. Although the load cycle is directly induced into the structure, instead of being the result of the constraint preventing the thermal expansion, the positive correlation between the natural frequency values and the load/axial stress on the specimen is confirmed. For the same load magnitudes in test T3, the natural frequency values measured in T4 were lower, likely due to the deterioration induced by the previous thermal cycle. Indeed, after T3, the values of the frequencies did not return to their initial levels when measured at the same reference ambient temperature. The linear relationship between frequencies and load, independent of the loading or unloading path, as identified in T3, is confirmed. However, in T4, unlike the thermal cycle, the load cycle did not cause further deterioration as the natural frequencies recovered their initial values at the end of the test.

## 4. Discussion

The present paper aims to experimentally investigate the modal behaviour of a brick masonry wall when exposed to changes in thermal load under expected operational conditions and various realistic loading and boundary conditions. A comprehensive experimental programme involving a 540 mm × 560 mm × 100 mm single-leaf wall was implemented. Four tests were conducted, comprising two thermal cycles affecting the specimen clamped at the base and free to expand (T1, T2); a thermal cycle affecting the specimen clamped at the base and constrained at the top to prevent expansion along the vertical direction (T3); and a load cycle applied at the top of the specimen clamped at the base under constant environmental conditions (T4). T1 and T2 aimed to investigate the effect of the temperature cycles on the masonry constituents and the final composite specimen in terms of the alteration of the modal properties under constant loading. T4 aimed to investigate the effect of precompression under constant temperature and relative humidity. T3 aimed to investigate the combination of the two phenomena, where the variation in the environmental parameters induces a change in the precompression, simulating the interaction among structural elements in a real-scale masonry structure under different temperature variations.

Tests T1 and T2 showed significant alterations induced by the thermal cycles despite the absence of visible signs of distress on the specimen. This result confirmed previous studies in which the deterioration mainly happened at the microstructure level. The alteration was characterised by a reduction in natural frequencies for increasing temperature, which was not fully recovered at the end of the cycle, indicating a residual stiffness reduction at the material level. On the other hand, T4 demonstrated that the natural frequencies increase with precompression. Within the investigated range, the natural frequencies returned to their initial value by the end of the cycle. When the two phenomena were combined, and the temperature variation induced an increase in load, the effect of the precompression appeared to govern the response of the specimen, increasing natural frequencies.

Nonetheless, a deterioration caused by the thermal cycle at the microstructural level, coupled with a reduction in the level of compression, emerged as a permanent reduction in natural frequencies at the end of the cycle compared to the initial values.

The promising experimental results obtained here suggest that the overall direct relationship between natural frequencies and temperature observed during long-term monitoring of masonry structures is likely due to the interaction within the structure. By preventing expansion, this interaction leads to the closure of micro-cracks and consequent stiffening despite the well-known negative effect of temperature on stiffness. However, in the present experimental programme, this interaction has been simulated through external controlled constraints. Therefore, investigating more complex three-dimensional masonry assemblies is a necessary future scope to study realistic interactions under varying temperature conditions. Furthermore, these first preliminary results pave the way to plan subsequent systematic tests on different structural types and build a database for testing and applying a FE numerical procedure investigated by the authors to calculate the frequencies of masonry structures in the presence of thermal loads consisting of a linear perturbation analysis which evaluates a structure’s dynamic properties by considering the non-linear behaviour of the constituent material and, therefore, the presence of fractures and damage, making finite element analysis a good practice in terms of structural health monitoring for baseline comparisons, simulating real-world conditions and the detection and localisation of damage.

## Figures and Tables

**Table 1:** Examples of variations in the frequencies of masonry structures as the temperature varies reported in the literature.

Author	Structures Typology	Difference Between the Maximum and Minimum Temperature Recorded [°C]	Maximum First Frequency Variation [%]	Maximum Second Frequency Variation [%]
Azzara et al. [10]	S. Frediano bell tower	38	5.42	6.50
Azzara et al. [10]	Clock tower	20	3.65	3.40
Barsocchi et al. [14]	Matilde donjon	36	7.35	11.28
Gentile et al. [12]	Gabbia tower	47	14.58	12.53
Ubertini et al. [13]	San Pietro bell tower	30	3.72	4.13
Gentile et al. [15]	Milan Cathedral	30	2.58	6.69
Kita et al. [16]	Consoli Palace	30	6.00	19.34

**Table 2:** Experimental sequence carried out on the masonry panel.

Test Number	Execution Date	Reference Temp. q_0_ [°C]	Boundary Conditions	Heaters	Load Cells
T1	9 October 2023	23.27	Clamped at the base	1	0
T2	10 October 2023	23.30	Clamped at the base	1	0
T3	11 October 2023	23.74	Clamped at the base and top Z-displacement prevented	2	2
T4	13 October 2023	23.97	Clamped at the base and load applied to the top	0	2

## Data Availability

Data will be made available upon reasonable request to the authors.

## Appendix A

**Table A1:** Test T1: the mock-up’s first three frequencies fi (q) and the corresponding average temperature values recorded at the exposed face S2 (TH5-TH7), unexposed face S1 (TH1-TH2-TH3), and in the specimen core (TH4-TH6) during the test.

Time [Minutes]	Mean TH1-TH2-TH3 [°C]	Mean TH5-TH7 [°C]	Mean TH4-TH6 [°C]	f_1_ [Hz]	f_2_ [Hz]	f_3_ [Hz]
0	23.31	23.28	23.19	43.06	140.76	148.27
74	25.43	61.52	30.07	41.79	137.36	145.94
114	30.92	39.82	38.35	41.87	138.10	145.85
155	31.64	33.06	33.87	42.02	138.50	145.92
175	30.85	31.61	32.47	42.10	138.38	145.71
207	29.61	29.89	30.66	42.16	138.60	145.61
237	28.70	28.64	29.30	42.17	139.16	145.84
Minimum frequency f_i,min_ [Hz]				41.79	137.36	145.61
Δf = (f_i0_ − f_i,min_)/f_i0_ [%]				2.95	2.41	1.79

**Table A2:** Test T2: the specimen’s first three natural frequencies f_i_ (i = 1…3) and the corresponding mean temperature values at the exposed face S2 (TH5-TH7-TH8), unexposed face S1 (TH1-TH2-TH3), and in the specimen core (TH4-TH6) during the test.

Time [Minutes]	Mean TH1-TH2-TH3 [°C]	Mean TH5-TH7-TH8 [°C]	Mean TH4-TH6 [°C]	f_1_ [Hz]	f_2_ [Hz]	f_3_ [Hz]
0	23.27	23.40	23.23	42.32	138.86	146.55
48	23.47	63.50	28.49	41.60	137.31	145.56
84	31.27	93.42	58.02	40.39	135.20	142.87
114	39.74	52.76	55.75	39.73	134.53	141.47
168	44.00	45.55	47.21	40.34	135.25	141.03
187	40.55	41.31	45.64	40.52	135.50	141.42
230	36.13	36.36	40.00	40.84	135.90	144.04
247	35.03	35.08	38.21	40.91	136.07	144.82
294	29.58	29.70	30.90	41.24	136.63	146.02
Minimum frequency f_i,min_ [Hz]				39.73	134.53	141.03
Δf = (f_i0_ − f_i,min_)/f_i0_ [%]				6.10	3.12	3.77
	23.68 *	23.86 *	23.67 *	41.56 *	137.26 *	144.902 *

* The mock-up’s temperatures and frequencies measured the day after before performing the T3 test.

**Table A3:** Test T1: the MAC values (MAC_1_, MAC_2_, MAC_3_) calculated between the specimen’s first three mode shapes ϕ_i_ and the corresponding ones ϕ_i0_ at the reference temperature.

Time [Minutes]	Mean TH1-TH2-TH3 [°C]	Mean TH5-TH7 [°C]	Mean TH4-TH6 [°C]	MAC_1_	MAC_2_	MAC_1_
0	23.31	23.28	23.19	1.00	1.00	1.00
74	25.43	61.52	30.07	1.00	0.99	1.00
114	30.92	39.82	38.35	1.00	0.99	0.97
155	31.64	33.06	33.87	1.00	0.99	0.98
175	30.85	31.61	32.47	1.00	0.99	0.99
207	29.61	29.89	30.66	1.00	0.99	1.00
237	28.70	28.64	29.30	1.00	0.99	0.96
Minimum MAC				1.00	0.99	0.96

**Table A4:** Test T2: the MAC values (MAC_1_, MAC_2_, MAC_3_) calculated between the specimen’s first three mode shapes ϕ_i_ and the corresponding ones ϕ_i0_ at the reference temperature.

Time [Minutes]	Mean TH1-TH2-TH3 [°C]	Mean TH5-TH7 [°C]	Mean TH4-TH6 [°C]	MAC_1_	MAC_2_	MAC_3_
0	23.27	23.40	23.23	1.00	1.00	1.00
48	23.47	63.50	28.49	1.00	1.00	1.00
84	31.27	93.42	58.02	1.00	0.99	1.00
114	39.74	52.76	55.75	1.00	0.97	0.96
168	44.00	45.55	47.21	1.00	0.99	1.00
187	40.55	41.31	45.64	1.00	0.98	0.98
230	36.13	36.36	40.00	1.00	0.98	0.95
247	35.03	35.08	38.21	1.00	0.98	0.98
294	29.58	29.70	30.90	1.00	0.97	0.96
MAC minimum				1.00	0.97	0.95

**Table A5:** Test T3: the mock-up’s frequencies and the corresponding average temperature values at the exposed face S2 (TH5-TH7-TH8), unexposed face S1 (TH1-TH2-TH3), and in the specimen core (TH4-TH6); the MAC values calculated between the specimen’s mode shape ϕ_i_ and the corresponding one ϕ_i0_ at the reference temperature; the reaction force measured by the load cells.

Time [Minutes]	Mean TH1-TH2-TH3 [°C]	Mean TH5-TH7-TH8 [°C]	Mean TH4-TH6 [°C]	Load Cell [kN]	f_1_ [Hz]	f_2_ [Hz]	MAC_1_	MAC_2_
36	23.74	24.02	23.75	5.14	36.66	117.27	1.00	1.00
101	42.36	39.19	36.82	6.40	37.53	118.96	1.00	1.00
127	35.56	34.94	38.02	5.94	37.38	118.10	1.00	0.99
140	34.61	34.25	38.00	5.72	37.07	118.00	1.00	0.98
178	32.27	32.40	34.41	5.04	36.55	117.69	1.00	0.99
206	31.18	31.16	32.77	4.71	36.32	117.40	1.00	0.99
323	27.58	27.67	28.33	3.90	36.04	116.73	1.00	0.98
	23.64 *	23.64 *	23.52 *	5.34 *	36.58 *	114.42 *	23.64 *	23.64 *
Maximum frequency f_i,max_ [Hz]					37.53	118.96	--	--
Δf = (f_i,max_ − f_i0_)/f_i0_ [%]					2.37	1.44	--	--
Minimum frequency f_i,min_ [Hz]					36.04	116.73	--	--
Δf = (f_i0_ − f_i,min_)/f_i0_ [%]					1.68	0.46	--	--
Minimum MAC					--	--	1.00	0.98

* The mock-up’s temperatures and frequencies measured the day after before performing the T3 test.

**Table A6:** Test T4: the mock-up’s frequencies fi and the corresponding value of the load applied; the MAC values calculated between the specimen’s mode shape ϕ_i_ and the corresponding one ϕ_i0_ at the beginning of the test.

Time [Minutes]	Load Cell [kN]	f_1_ [Hz]	f_2_ [Hz]	MAC_1_	MAC_2_
20	0.12	25.83	114.72	1.00	1.00
118	0.98	28.05	118.41	1.00	1.00
128	1.98	28.63	118.78	1.00	0.99
159	2.92	29.24	119.56	1.00	0.98
174	3.99	31.26	120.78	1.00	0.98
184	5.43	33.87	122.92	1.00	0.99
196	6.03	34.88	123.65	1.00	0.98
256	7.31	36.22	125.21	1.00	0.97
265	4.00	32.56	122.00	1.00	0.99
273	2.08	28.49	120.06	1.00	0.99
319	0.12	26.18	--	1.00	--
Maximum frequency f_max_ [Hz]		36.22	125.21		
Maximum increment Δf = (f_max_ − f_0_)/f_0_ [%]		40.25	9.09		
